# Tah1 helix-swap dimerization prevents mixed Hsp90 co-chaperone complexes

**DOI:** 10.1107/S1399004715004551

**Published:** 2015-04-25

**Authors:** Rhodri M. L. Morgan, Mohinder Pal, S. Mark Roe, Laurence H. Pearl, Chrisostomos Prodromou

**Affiliations:** a Genome Damage and Stability Centre, School of Life Sciences, University of Sussex, Falmer, Brighton BN1 9RQ, England

**Keywords:** Tah1, Hsp90, co-chaperones

## Abstract

Specific co-chaperone adaptors facilitate the recruitment of client proteins to the Hsp90 system. Tah1 binds the C-terminal conserved MEEVD motif of Hsp90, thus linking an eclectic set of client proteins to the R2TP complex for their assembly and regulation by Hsp90. Rather than the normal complement of seven α-helices seen in other tetratricopeptide repeat (TPR) domains, Tah1 unusually consists of the first five only. Consequently, the methionine of the MEEVD peptide remains exposed to solvent when bound by Tah1. In solution Tah1 appears to be predominantly monomeric, and recent structures have failed to explain how Tah1 appears to prevent the formation of mixed TPR domain-containing complexes such as Cpr6–(Hsp90)_2_–Tah1. To understand this further, the crystal structure of Tah1 in complex with the MEEVD peptide of Hsp90 was determined, which shows a helix swap involving the fifth α-helix between two adjacently bound Tah1 molecules. Dimerization of Tah1 restores the normal binding environment of the bound Hsp90 methionine residue by reconstituting a TPR binding site similar to that in seven-helix-containing TPR domain proteins. Dimerization also explains how other monomeric TPR-domain proteins are excluded from forming inappropriate mixed co-chaperone complexes.

## Abstract 2

A helix swap involving the fifth helix between two adjacently bound Tah1 molecules restores the normal binding environment of the conserved MEEVD peptide of Hsp90. Dimerization also explains how other monomeric TPR-domain proteins are excluded from forming inappropriate mixed co-chaperone complexes with Hsp90 and Tah1.

## 1. Introduction   

The R2TP complex is responsible for the cellular stabilization and assembly of a specific set of proteins and macromolecular complexes (Te *et al.*, 2007 ▶; Zhao *et al.*, 2005 ▶), including RNA polymerase II (Boulon *et al.*, 2010 ▶; Forget *et al.*, 2010 ▶), small nucleolar ribonucleoproteins (snoRNPs; Gonzales *et al.*, 2005 ▶; Kakihara & Houry, 2012 ▶; Kurokawa *et al.*, 2008 ▶; Samarsky *et al.*, 1998 ▶) and phosphatidylinositol-3-kinase-like kinases (PIKKs) such as mTOR and SMG1 (Hořejší *et al.*, 2010 ▶; Takai *et al.*, 2010 ▶). The R2TP complex consists of the AAA+ ATPase Rvb1 and Rvb2 heterododecamer complex (RUVBL1 and RUVBL2 in humans), a tetratricopeptide (TPR)-containing protein (Tah1, or RPAP3 in humans and Spag/Spaghetti in *Drosophila*) and Pih1 (also known as NOP17) (Kakihara & Houry, 2012 ▶; Te *et al.*, 2007 ▶). The recruitment of Hsp90 by the R2TP complex is essential for its biological role (Pal *et al.*, 2014 ▶; Boulon *et al.*, 2010 ▶; Izumi *et al.*, 2012 ▶; Takai *et al.*, 2010 ▶). In yeast, Tah1 acts as the direct link between the R2TP complex and Hsp90 by binding the C-terminal conserved MEEVD peptide motif of the chaperone and the C-terminal region of Pih1 (Eckert *et al.*, 2010 ▶). In metazoa, the interaction of Pih1 with Hsp90 is mediated by Spag/RPAP3 (Itsuki *et al.*, 2008 ▶), a larger protein containing two N-terminal TPR domains as well as additional domains of unknown function.

Recently, NMR and crystal structures (Pal *et al.*, 2014 ▶; Back *et al.*, 2013 ▶; Jiménez *et al.*, 2012 ▶) revealed that Tah1 consists of five α-helices, rather than the typical seven α-helices observed in other TPR domains that bind the Hsp90 MEEVD peptide. A virtually identical twisted Hsp90 peptide conformation and set of interactions is observed in these structures and those of CHIP and AIP (Morgan *et al.*, 2012 ▶; Zhang *et al.*, 2005 ▶; Pal *et al.*, 2014 ▶; Back *et al.*, 2013 ▶), even though Tah1 lacks the two C-terminal α-helices (6 and 7). Consequently, the hydrophobic pocket that is normally formed by amino-acid residues from α-helices 5 and 7, as seen in AIP and CHIP, is incomplete and the methionine of the MEEVD peptide remains exposed to the solvent. Curiously, the TPR1 and TPR2 domains of Spag/RPAP3 appear to possess the normal complement of α-helices such that the methionine-accepting hydrophobic pocket is intact (Pal *et al.*, 2014 ▶). This suggests that the full molecular details by which the MEEVD peptide of Hsp90 binds the yeast Tah1 protein have still not been fully elucidated.

In solution, Tah1 appears to be predominantly monomeric and binds Hsp90 as a 1:1 stoichiometric (two Tah1 monomers:one Hsp90 dimer) complex (Pal *et al.*, 2014 ▶). This complex excludes interaction with Cpr6 and thus prevents the formation of a Cpr6–(Hsp90)_2_–Tah1 mixed co-chaperone complex (Pal *et al.*, 2014 ▶). However, the mechanism by which this occurs remains elusive. The exclusion of co-chaperones that are not normally part of a specific Hsp90 client–protein complex is fundamentally essential for the proper activation of client proteins. Homodimerization of a specific TPR-domain protein following Hsp90 binding is a possible mechanism by which other monomeric TPR-domain proteins would be excluded from simultaneously binding. Homodimerization would thus allow both MEEVD sites of Hsp90 to be simultaneously occupied. The fact that Tah1 is weakly dimeric in solution (Pal *et al.*, 2014 ▶) raises this as a distinct possibility, although to date direct structural dimerization of Tah1 has not been observed (Back *et al.*, 2013 ▶; Jiménez *et al.*, 2012 ▶; Pal *et al.*, 2014 ▶). Theoretically, dimerization of Tah1 could also provide the extra shielding from solvent of the methionine of the bound MEEVD peptide of Hsp90.

To address these questions, we have obtained a new crystal form of Tah1 in complex with the EDASRMEEVD peptide of Hsp90 that reveals a swap between α-helix 5 of two adjacently Hsp90-bound Tah1 molecules. The dimerization of Tah1 reconstitutes the typical MEEVD binding mode observed with seven-helix-containing TPR domains such as AIP and CHIP (Morgan *et al.*, 2012 ▶; Zhang *et al.*, 2005 ▶) and explains how other monomeric TPR proteins are excluded from binding simultaneously.

## 2. Methods   

### 2.1. Protein purification   

For selenomethionine (SeMet) labelling, *Escherichia coli* Rosetta DE3 cells containing the appropriate expression vector were grown in minimal medium supplemented with SeMet. Synthetic full-length yeast Tah1 was expressed from pRSET-A. Tah1 was purified by Talon affinity chromatography (Clontech, Oxford, England) equilibrated in 20 m*M* Tris pH 7.5 containing 100 m*M* NaCl and eluted with the same buffer but containing 500 m*M* imidazole at pH 7.0. The protein was then concentrated using Vivaspin concentrators (5000 Da molecular-weight cutoff) and subjected to Superdex 75 HR gel-filtration chromatography equilibrated in 20 m*M* Tris pH 7.5 containing 500 m*M* NaCl, 0.5 m*M* TCEP. Pure Tah1 was dialyzed in 10 m*M* HEPES pH 7.5, 140 m*M* NaCl, 0.5 m*M* TCEP and then concentrated to 10 mg ml^−1^ in the presence of 5 m*M* Hsp90 peptide (EDASRMEEVD) and stored frozen at −20°C. Purification of AIP was as previously described (Morgan *et al.*, 2012 ▶).

### 2.2. Crystallography   

The Tah1–EDASRMEEVD complex was crystallized using the sitting-drop method with protein at 10 mg ml^−1^ in 140 m*M* NaCl, 10 m*M* Na HEPES pH 7.8, 0.5 m*M* TCEP against wells containing 100 m*M* MES pH 6.0, 200 m*M* ammonium chloride, 20% PEG 6000 at 4°C. Crystals were harvested by successive transfer into crystallization buffer with increasing ethylene glycol content to 30% and then flash-cooled in liquid nitrogen. Diffraction data were collected from crystals cooled to 100 K on an in-house Rigaku MicroMax-007 HF generator at the Cu wavelength. Data were processed with *iMosflm* (Battye *et al.*, 2011 ▶) and the asymmetric unit contents were estimated using the Matthews coefficient (*CCP*4 suite; Krissinel *et al.*, 2004 ▶). The Tah1–EDASRMEEVD structure was solved using *Phaser* (*CCP*4) with the model of Tah1 from the EDASRMEEVD–Tah1–Pih1p^187–344^ complex (Pal *et al.*, 2014 ▶). The structure of Tah1–EDASRMEEVD was refined with *REFMAC* 5.7 (Murshudov *et al.*, 2011 ▶) and manual rebuilding was performed in *Coot* (Emsley & Cowtan, 2004 ▶). Only the SRMEEVD residues of the Hsp90 peptide were visible. The structure was deposited in the PDB (PDB entry 4cgq) and was displayed using *PyMOL* (Schrödinger, USA).

### 2.3. Pulldowns and gel filtration   

Increasing concentrations of AIP^172–315^ or full-length AIP and 70 µ*M* Flag-Tah1 and Hsp90^600–709^ were incubated in 20 m*M* HEPES pH 7.5, 150 m*M* NaCl, 10% glycerol, 0.5% IGEPAL, 2 m*M* EDTA. Subsequently, 30 µl anti-Flag M2 magnetic beads (Sigma–Aldrich) were added. The beads were then washed and the eluate was subjected to SDS–PAGE. Superdex 75 gel-filtration chromatography was used to determine the presence of dimeric Tah1. The column was equilibrated with 20 m*M* Tris–HCl, 140 m*M* NaCl pH 7.5, 0.5 m*M* TCEP and calibrated using standard molecular-weight markers (bovine serum albumin, 67 kDa; ovalbumin, 44 kDa; carbonic anhydrase, 29 kDa; ribonuclease, 13.7 kDa; aprotinin, 6.5 kDa).

## 3. Results   

### 3.1. Structure of the Tah1–Hsp90 MEEVD complex   

**Figure 1:**
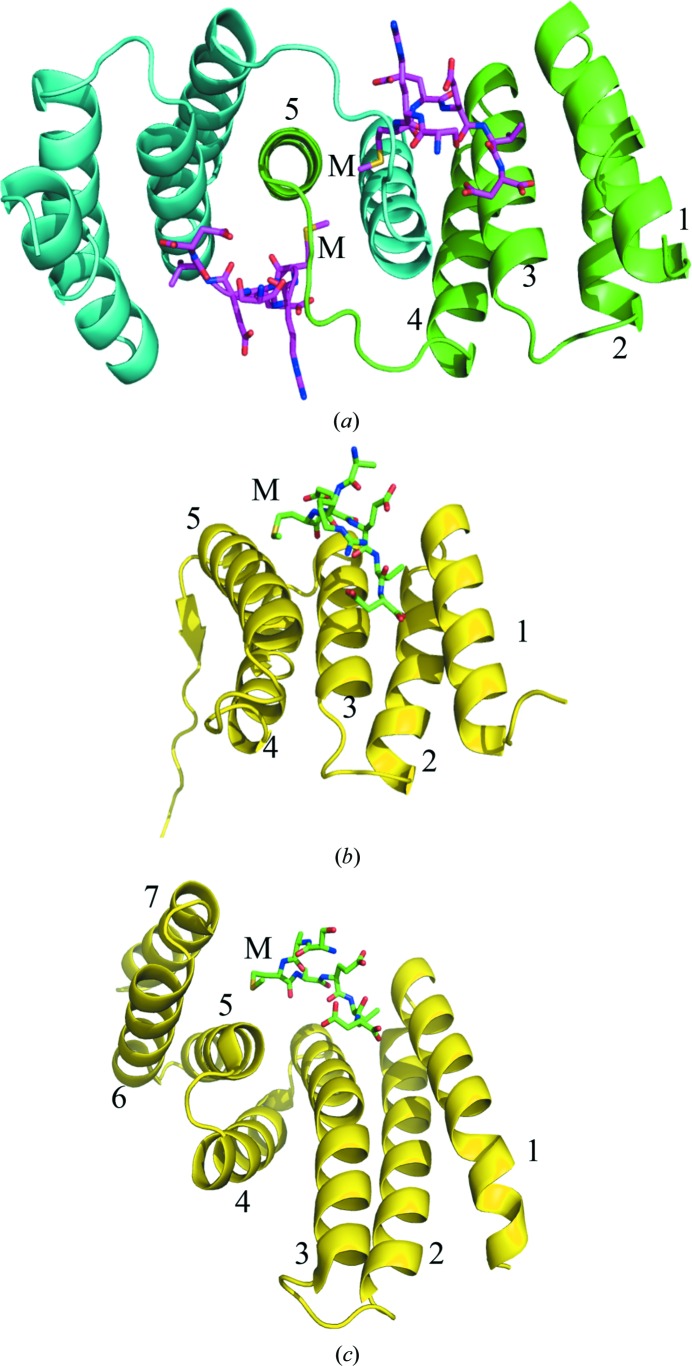
Structures of Tah1 and AIP. (*a*) Secondary-structure cartoon of the Tah1 TPR-domain dimer (cyan and green). The fifth helix of one TPR domain intercalates between the fourth and fifth helices of the other, generating an effective six-helix two-TPR-domain structure. The bound Hsp90 C-­terminal peptides are shown as magenta sticks. Helices are numbered 1–5 and M represents the conserved methionine residue of the EDASRMEEVD peptide (only SRMEEVD was visible), now bound in a hydrophobic pocket formed by amino-acid residues Val74, Ser78, Lys79, Gln81, Tyr82 and Arg83 of Tah1. (*b*) Secondary-structure cartoon of the monomeric TPR domain of Tah1 (yellow) with bound SRMEEVD peptide derived from the C-terminus of Hsp90 (green sticks). Helices are numbered 1–5 and M represents the conserved methionine residue of the EDASRMEEVD peptide (only SRMEEVD was visible). (*c*) Secondary-structure cartoon of the TPR domain of AIP (yellow) bound to the SRMEEVD peptide derived from the C-terminus of Hsp90 (green sticks). Helices are numbered 1–5 and M represents the conserved methionine residue of the EDASRMEEVD peptide (only SRMEEVD was visible).

**Figure 2:**
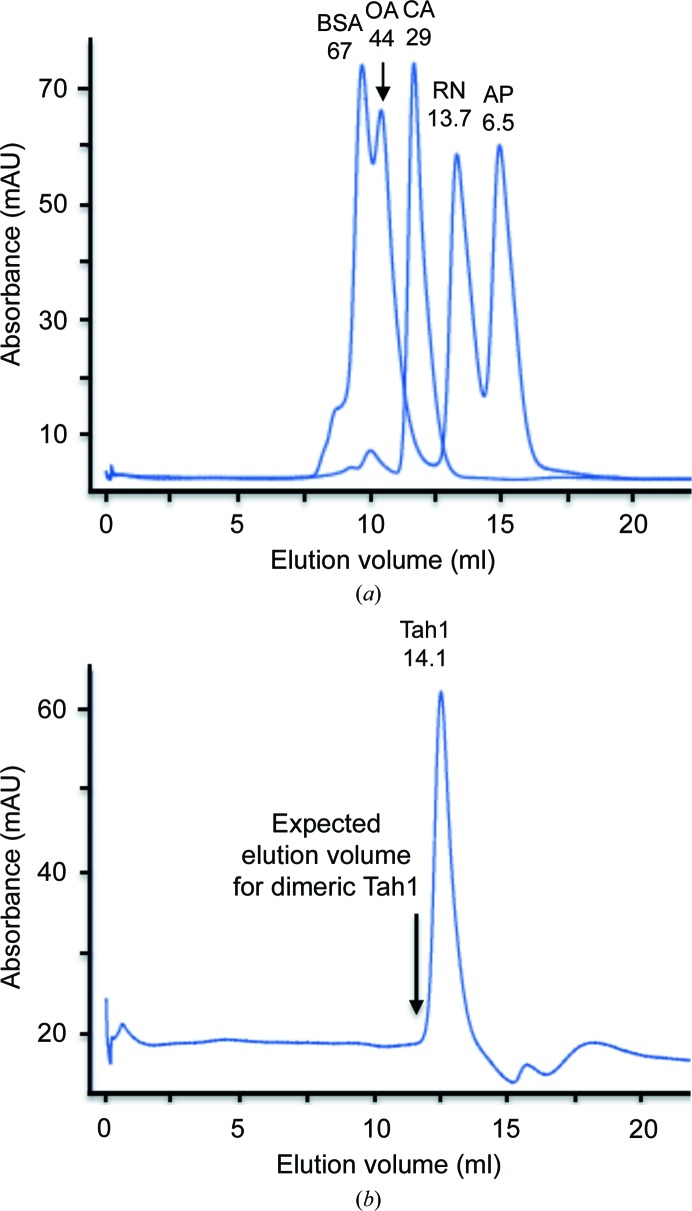
Gel-filtration analysis of Tah1. (*a*) Calibration of the Superdex 75 HR gel-filtration column with protein standards. BSA, bovine serum albumin; OA, ovalbumin; CA, carbonic anhydrase; RN, ribonuclease A; AP, aprotinin. The molecular mass of each standard protein is indicated in kDa. (*b*) Gel-filtration profile of Tah1. Monomeric Tah1 clearly elutes with a molecular weight of 14.1 kDa, with no indication of a shoulder or a second peak that might represent the presence of dimeric Tah1. This suggests that Tah1 is mainly monomeric in solution. The arrow indicates the expected elution volume of dimeric Tah1. mAU, milliabsorbance units.

The structure of a complex between the TPR domain of Tah1 and the Hsp90 peptide EDASRMEEVD (residues 1–7 of which, S^1^RMEEVD^7^, are the only residues visible in the structure) was solved by single-wavelength anomalous diffraction (see §[Sec sec2]2) and refined to 2 Å resolution (Table 1 ▶). 97.4% (299 out of 307) of all residues were in favoured (98%) regions and 100.0% (307 out of 307) of all residues were in allowed (>99.8%) regions (Supplementary Fig. S1). The structure reveals that two Tah1 molecules form an intimate dimer by symmetrical exchange of their C-terminal α-helices (Fig. 1 ▶ *a*), in contrast to the structures previously observed for Tah1 (Pal *et al.*, 2014 ▶; Back *et al.*, 2013 ▶; Jiménez *et al.*, 2012 ▶; Fig. 1 ▶ *b*) and other TPR-domain proteins such as AIP (Fig. 1 ▶ *c*), and supports the observation that Tah1 appears to be a weak dimer in solution (Pal *et al.*, 2014 ▶). Weak dimer formation by Tah1 was confirmed by gel-filtration chromatography, which showed that Tah1 was predominantly monomeric in solution with no significant dimer peak (Fig. 2 ▶).

**Figure 3:**
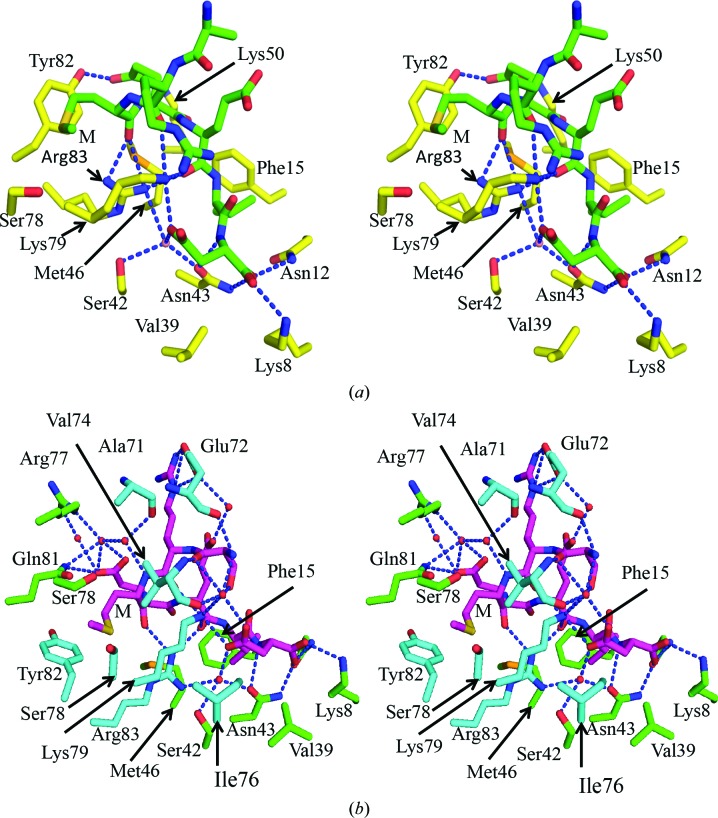
Stereo *PyMOL* images of the interactions of the Hsp90 peptide with monomeric and dimeric Tah1. (*a*) Detail of interactions of the monomeric Tah1 TPR domain and the Hsp90 C-terminal SRMEEVD peptide. The Hsp90 peptide binds with a compacted conformation stabilized by interaction of the side chain of Glu4 with the side chains of Lys50 and Tyr82 of Tah1. The backbone carbonyl of Glu5 is stabilized by interactions with the side chains of Lys79 and Arg83 of Tah1. The peptide carbonyl of Met3 is also stabilized by interaction with the side chain of Arg83. The α-carboxyl and carboxylate side chain of Asp7 is bound by a ‘carboxylate clamp’ formed by residues Lys8, Asn12, Asn43 and Lys79 of Tah1. The side chain of Met3 in the Hsp90 peptide packs against Tyr82, but is far more exposed than seen in other Hsp90–TPR domain complexes. M represents the conserved methionine residue of the EDASRMEEVD peptide (only SRMEEVD was visible). (*b*) Detail of the interactions of the Hsp90 C-terminal SRMEEVD peptide with the helix-swapped Tah1 TPR domain dimer. Essentially the same set of interactions occurs as in the monomeric TPR (Fig. 3 ▶ *a*), but with residues from both TPR domains in the dimer. Additional dimer-specific polar interactions occur between Glu4 of the Hsp90 peptide and Gln81 of Tah1 and between the peptide backbones of Glu72 and Val74 of Tah1 and the N-terminal end of the peptide, while Met3 becomes buried in a hydrophobic pocket formed by Tah1 residues Val74, Ser78, Lys79, Gln81, Tyr82 and Arg83. M represents the conserved methionine residue of the EDASRMEEVD peptide (only SRMEEVD was visible).

**Figure 4:**
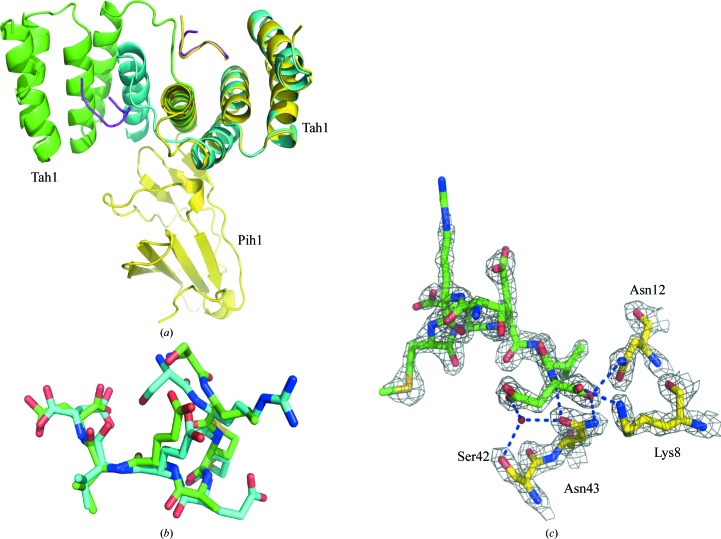
Hsp90 peptide conformations. (*a*) Cartoon superimposition of the Tah1–Pih1 (yellow) structure and the Tah1 dimeric structure (green and cyan) showing that the bound Hsp90 peptides [shown as tubes in magenta for the dimer structure and yellow for the Tah1 (monomer)–Pih structure] are bound in essentially the same conformation. (*b*) Superimposition of the Tah1-bound (cyan) and the AIP-bound (green) EDASRMEEVD Hsp90 peptides (only SRMEEVD is visible) showing that they bind in essentially the same conformation. (*c*) Cartoon stick model showing the carboxylate-clamp interactions between the Hsp90 peptide EDASRMEEVD (green; only SRMEEVD is visible) and Tah1 amino-acid residues (yellow).

**Figure 5:**
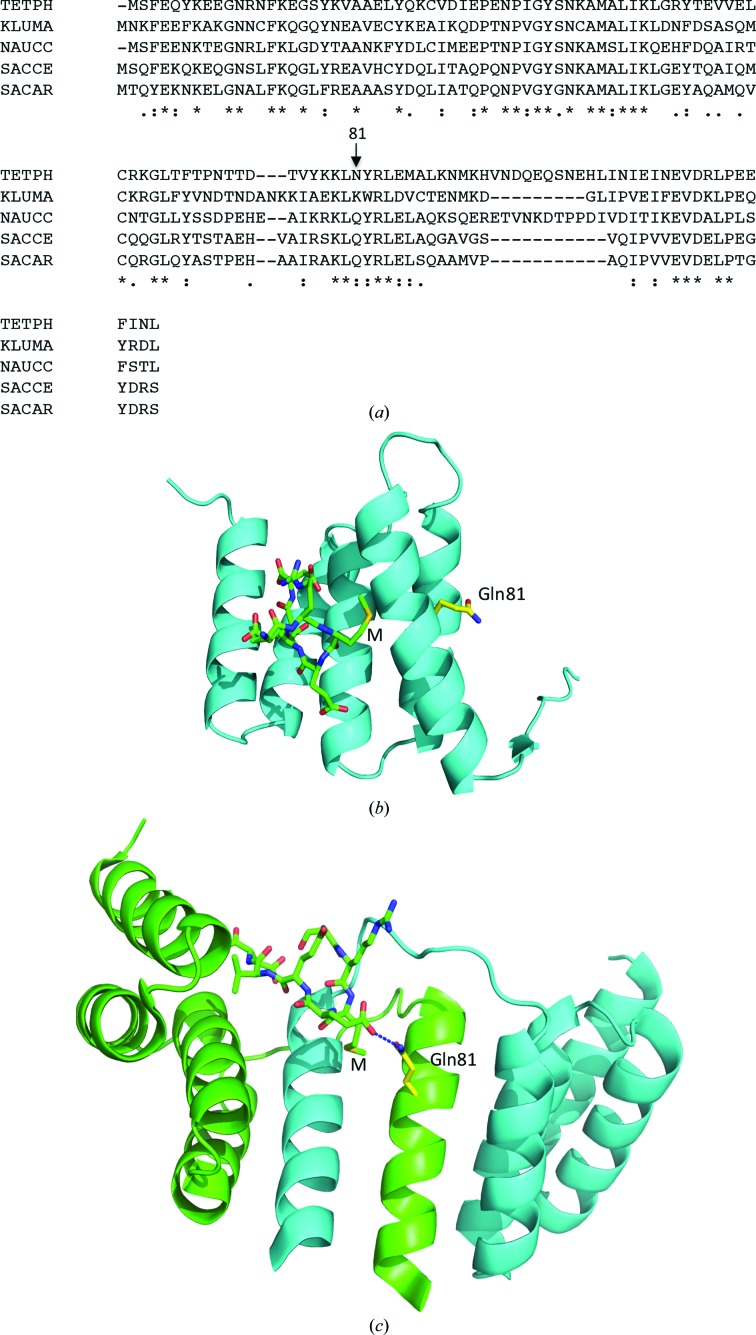
Conservation of amino-acid residues of Tah1. (*a*) Sequence alignment showing conserved residues in Tah1 sequences. Position 81 is indicated. TETPH, *Tetrapisispora phaffii*; KLUMA, *Kluyveromyces marxianus*; NAUCC, *Naumovozyma castellii*; SACCE, *Saccharomyces cerevisiae*; SACAR, *Saccharomyces arboricola*. Asterisks and dots below the alignment signify the degree of conservation. (*b*) Monomeric Tah1 showing the solvent-exposed position of Gln81. M represents the methionine residue of the MEEVD peptide. (*c*) Dimeric Tah1 showing the interactions made by the Gln81 residues of Tah1. M represents the methionine residue of the MEEVD peptide.

The dimeric Tah1 structure reveals that the α-helix swap seen between two molecules of Tah1 reconstructs the hydrophobic binding pocket that accepts the conserved methionine of the SRMEEVD peptide (compare Figs. 3 ▶ *a* and 3 ▶ *b*). The Hsp90 peptide essentially adopts the same conformation as previously seen in Tah1 (monomeric) and AIP structures, including the important carboxylate-clamp interaction (Fig. 4 ▶). Consequently, the interactions made in previous Tah1 structures are retained in the dimer complex (compare Figs. 3 ▶ *a* and 3 ▶ *b*; Pal *et al.*, 2014 ▶). However, some additional interactions are seen in the dimeric structure. Interactions between the Hsp90 peptide and the Arg77, Lys79, Gln81, Tyr82 and Arg83 residues of Tah1 are provided by the swapped C-terminal α-helix from the other monomer (Fig. 3 ▶ *b*). Significantly, Gln81 not only makes a set of new interactions with the MEEVD peptide but also completes the hydrophobic pocket into which the methionine of the Hsp90 peptide binds. In Tah1 this position shows amino-acid residue conservation even though these residues are exposed to solvent in the monomeric structures (Fig. 5 ▶).

The formation of the swapped helix dimer sees the first turn of the fifth α-helix unravel to form an extended segment of polypeptide chain that allows swapping of the rest of the helix into the TPR structure of the other monomer. The residues forming this extended segment also provide additional interactions with the Hsp90 peptide, including main-chain hydrogen bonds between the Glu72 and Val74 residues of Tah1 and the first residue (Ser1) of the bound peptide. The side chain of Val74 of Tah1 also packs against the side chain of Met3 in the Hsp90 peptide, thus fully enclosing it in a hydrophobic pocket (Fig. 3 ▶ *b*). The helix exchange observed in the dimerized five-helix TPR structure of Tah1 therefore restores the hydrophobic burial of the methionine side chain of the Hsp90 peptide, similar to that observed in seven-helix TPR domains.

### 3.2. The start of α-helix 5 of Tah1 is metastable   

**Figure 6:**
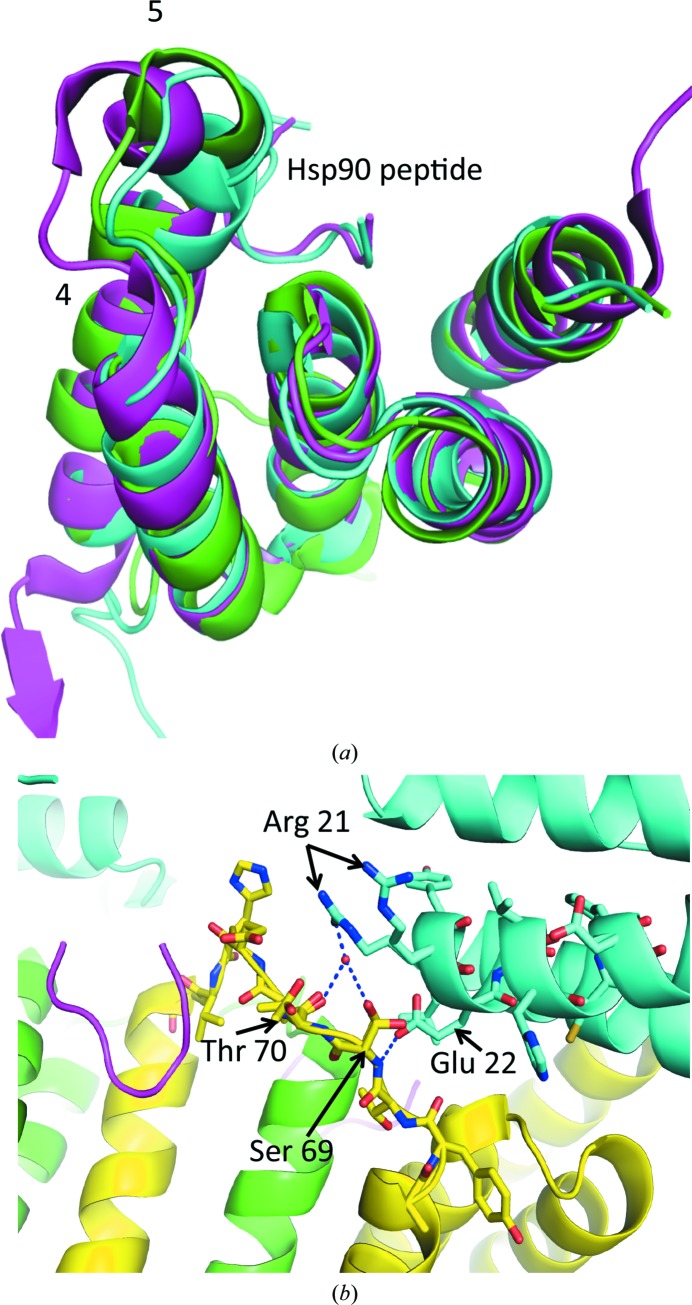
Superimposition of monomeric Tah1 structures. (*a*) Tah1 monomeric structures are superimposed. Magenta represents Tah1 from the structure of the Tah1–Pih complex (PDB entry 4cgu; Pal *et al.*, 2014 ▶), while green and cyan are solution structures (PDB entries 2lsu and 2lsv, respectively; Back *et al.*, 2013 ▶). Helices 5 and 4 are labelled. The tubes in magenta and cyan represent the bound Hsp90 peptide. (*b*) Crystallographic interface between the loop connecting α-helices 4 and 5 and an adjacent Tah1 monomer. Yellow and green represent the two halves of a Tah1 dimer in contact with an adjacent Tah1 molecule (shown in cyan). Hydrogen bonds are shown as dotted blue lines, while water molecules are shown as red spheres.

Fundamental to the formation of the Tah1 helix-swap dimer is the unravelling of the start of helix 5. Thus, we compared a number of monomeric Tah1 structures to understand whether this region was structurally variable (Pal *et al.*, 2014 ▶; Back *et al.*, 2013 ▶). Analysis showed that the MEEVD peptide of Hsp90 binds in essentially the same conformation and that the methionine is exposed to solvent in all cases (Fig. 6 ▶ *a*). However, the most divergent part of the structure, apart from the unstructured C-terminal extension of Tah1, was the start of α-helix 5 and the loop leading to it (Fig. 6 ▶ *a*). This analysis suggests that this region of Tah1 is probably metastable and might help to initiate the movement of α-helix 5 towards the formation of the helix-swapped state.

We next investigated whether the crystallization contacts between the loop connecting helices 4 and 5 of Tah1 and the other Tah1 molecules in the crystal lattice might have induced the helix-swapped conformation. The section of the fifth helix that unwinds (Thr70–Tyr74) is the same section as is involved in crystallization contacts. Specifically, the main-chain amide group of Ser69 makes a hydrogen-bond inter­action with the carbonyl group of Glu22 of a neighbouring molecule. This appears to be the only stable hydrogen-bond interaction. The side-chain hydroxyl group of Ser69 and the main-chain carbonyl group of Thr70 are also hydrogen-bonded, *via* a water-mediated interaction, to one of the guanidinium N atoms of Arg21 of the adjacent molecule. However, Arg21 and Ser69 are both seen to adopt alternate conformations, indicating that these are not very stable interactions (Fig. 6 ▶ *b*). It therefore appears that the interface between the loop connecting helices 4 and 5 and the neighbouring Tah1 molecule is rather weak. In contrast, the interactions formed between the loop and the bound MEEVD peptide are significantly more extensive and stable and include inter­actions with Ala71, Glu72 and Val74 (Fig. 3 ▶ *b*).

### 3.3. Tah1 binding excludes the binding of monomeric AIP   

**Figure 7:**
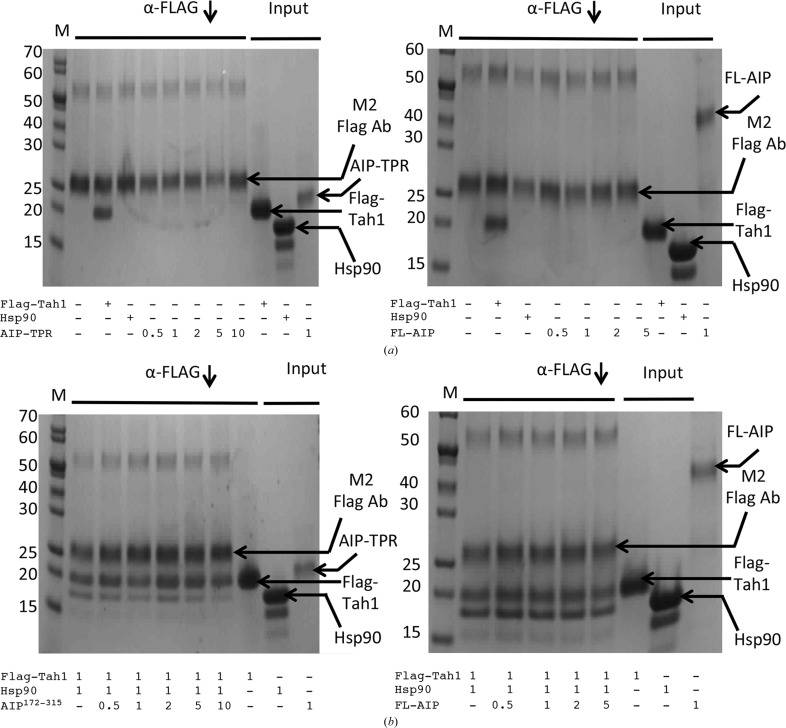
Dimerization of Tah1 prevents AIP binding. SDS–PAGE gels showing that Tah1 prevents the formation of a Tah1–cHsp90–AIP mixed complex. (*a*) Control pulldowns in the presence of AIP^172–315^ (left panel) or full-length AIP, Flag-Tah1 or cHsp90 alone (right panel). (*b*) Pulldowns with Flag-Tah1 in the presence of cHsp90 and increasing amounts of AIP^172–315^ (left panel) or full-length AIP (right panel). A fivefold to tenfold excess of either AIP^172–315^ or full-length AIP does not pull down with the Flag-Tah1–cHsp90 complex, indicating that a three-way complex with AIP does not form. Arrows indicate the positions of the various proteins used.

Previously, we had shown that Tah1 binding to Hsp90 could prevent the simultaneous binding of Cpr6 (Pal *et al.*, 2014 ▶). To further validate this, we also tested the ability of AIP (aryl hydrocarbon receptor-interacting protein), a monomeric TPR domain-containing protein, to form a mixed co-chaperone complex with Tah1. Tah1 binds with a slightly higher affinity than that of AIP (*K*_d_ = 5.9 and 13.3 µ*M*, respectively), so we used a 5–10 molar excess of AIP (both AIP^172–315^ and full-length AIP; Morgan *et al.*, 2012 ▶). We found that AIP did not pull down with any Tah1 by forming a mixed Tah1–Hsp90–AIP complex (Fig. 7 ▶). Thus, we conclude that dimerization of Tah1 following binding to Hsp90 appears to prevent the binding of AIP and Cpr6, as previously observed (Pal *et al.*, 2014 ▶).

### 3.4. Dimerization of Tah1 reconstitutes a more complete TPR domain that is compatible with the binding of Pih1   

**Figure 8:**
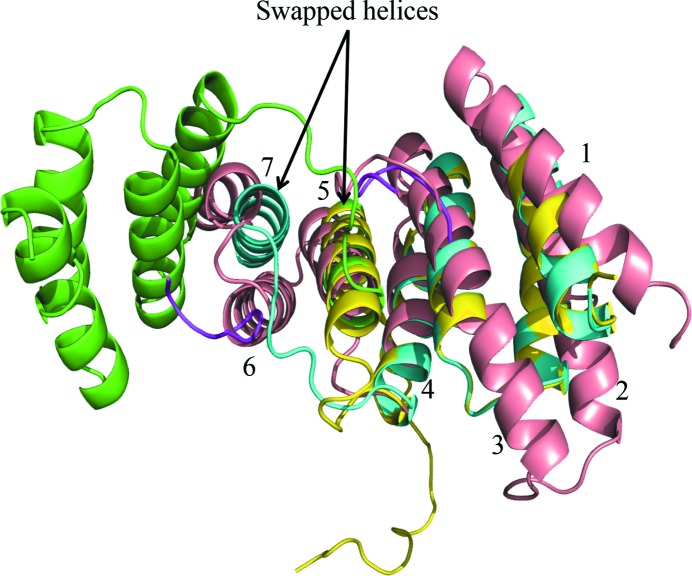
Dimerization of Tah1 reconstitutes a six-helix TPR domain. Superimposition of secondary-structure cartoons of dimeric Tah1 (green and cyan), monomeric Tah1 (yellow) and AIP (salmon). The helices of AIP are numbered 1–7. The swapped helices in the Tah1 dimer reconstruct the approximate position of helices 5 and 7 in the AIP structure.

When the Tah1 TPR domain from the structure of the Pih1–Tah1–Hsp90 peptide complex is superimposed on one monomer of the helix-swapped Tah1 dimer, the endogenous C-terminal helix from the former almost precisely overlays the exogenous C-terminal helix from the other Tah1 monomer in the dimeric structure (Fig. 8 ▶). This reconstitutes a more complete TPR domain, which although missing helix 6 (Fig. 8 ▶) maintains all of the essential contacts (compare Figs. 3 ▶ *a* and 3 ▶ *b*) required to satisfy the binding of the MEEVD peptide of Hsp90 (Pal *et al.*, 2014 ▶; Morgan *et al.*, 2012 ▶; Zhang *et al.*, 2005 ▶).

**Figure 9:**
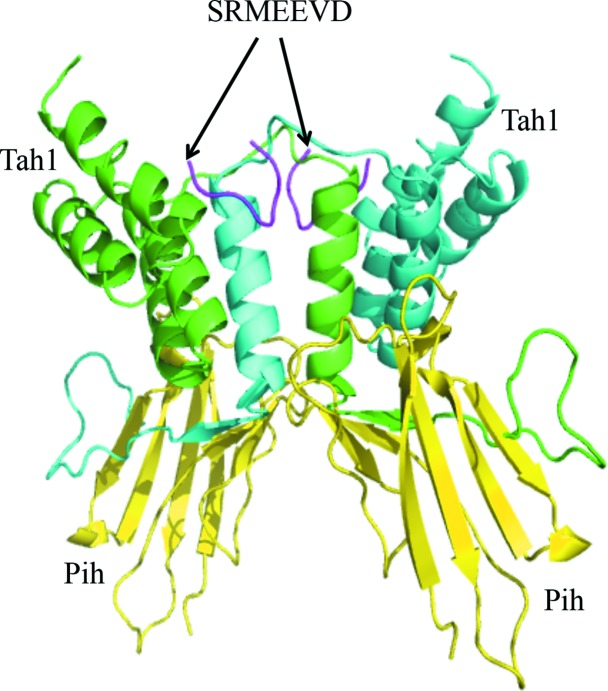
Dimerization of Tah1 permits Pih1 binding. Superimposition of secondary-structure cartoons of dimeric Tah1 (green and cyan) and Pih1 (yellow) bound to Tah1, showing that dimerization of Tah1 does not result in steric hindrance to the binding of Pih1. SRMEEVD represents the visible peptide of Hsp90 that was bound (magenta tubes).

Superimposition of Tah1 from the Tah1–Pih1 CS domain complex onto the dimeric Tah1 structure reveals no steric clash involving the Pih1 CS domain that might preclude each Tah1 bound to the C-terminus of Hsp90 from recruiting a Pih1 molecule (Fig. 9 ▶). This is consistent with results showing that Hsp90 binds Tah1 and preassembled Tah1–Pih1 complex not only with a 1:1 stoichiometry but also with similar affinity (*K*_d_ = 5.9 ± 0.3 and 4 ± 0.7 µ*M*, respectively; Pal *et al.*, 2014 ▶). The similar affinity and binding stoichiometry are consistent with the formation of an (Hsp90)_2_–(Tah1)_2_–(Pih1)_2_ complex in which the Tah1 molecules are dimerized.

## 4. Discussion   

The Hsp90 molecular-chaperone system is responsible for the assembly, stabilization and activation of a variety of proteins and complexes from protein kinases, including phosphatidyl­inositol 3-kinase-related kinases (PIKKs), to steroid hormone receptors, NLR innate immunity receptors and both viral and cellular DNA and RNA polymerases (Pearl *et al.*, 2008 ▶; Prodromou, 2012 ▶). Recruitment of client proteins to the Hsp90 system is mediated by specific co-chaperones that act as adaptors linking the client protein to Hsp90. For example, the Cdc37 co-chaperone recruits conventional protein kinases to Hsp90 (Vaughan *et al.*, 2006 ▶). During loading, the ATPase-coupled conformational cycle of the chaperone is halted as well as the kinase activity of the client protein (Siligardi *et al.*, 2002 ▶; Roe *et al.*, 2004 ▶; Polier *et al.*, 2013 ▶). Similarly, the dimeric TPR domain-containing protein HOP (Sti1 in yeast) delivers the steroid hormone–Hsp70 complex to Hsp90 while simultaneously silencing the ATPase activity of Hsp90. In contrast, PIKKs, RNA polymerase II and the snoRNPs and chromatin-remodelling complexes are recruited to Hsp90 by a far more complicated system involving a chain of protein interactions mediated by Tah1 (or Spag/RPAP3), which couples the R2TP complex to Hsp90 (Kakihara & Houry, 2012 ▶). In particular, the recruitment of PIKKs involves a far more protracted and potentially convoluted link between client protein and chaperone that utilizes the TTT complex (TEL2, TTI1 and TTI2; Pal *et al.*, 2014 ▶).

The interaction of Tah1 with Hsp90 is therefore crucial in recruiting Hsp90 into complex with R2TP-dependent client proteins. Here, we show that Tah1 undergoes a previously unobserved molecular rearrangement that results in a swap between the C-terminal α-helices of two adjacent Tah1 molecules. The intimate homodimer that is created reforms the hydrophobic binding pocket responsible for binding the conserved methionine residue of the MEEVD peptide of Hsp90. Ultimately, the helix swap allows the fifth helix of Tah1 to be involved in roles not only observed for helix 5 but also for helix 7 in conventional TPR domains such as AIP and CHIP (Morgan *et al.*, 2012 ▶; Zhang *et al.*, 2005 ▶). Exchange of secondary structures or even whole domains as part of dimer formation is a well known phenomenon (Liu & Eisenberg, 2002 ▶; Nagradova, 2002 ▶) and indeed plays a role in the N-terminal domain association that occurs as part of the ATPase-coupled conformational cycle of Hsp90 itself (Ali *et al.*, 2006 ▶).

Compelling evidence for the dimerization of Tah1 is provided by several observations. Significantly, there is strong amino-acid residue conservation at position 81 (Gln81 in *S. cerevisiae* Tah1) on the solvent-exposed surface of the fifth helix in the monomeric Tah1. The residue at this position varies from glutamine to asparagine or lysine, all of which could potentially interact with the bound Hsp90 peptide and simultaneously provide the shielding needed to complete the hydrophobic pocket. The fact that Gln81 is centrally important in the interaction with the MEEVD peptide while not disrupting the peptide-binding conformation, and simultaneously completes the hydrophobic binding pocket, is strong evidence that biologically Tah1 functions by dimerization. Ultimately, a prerequisite to the formation of a dimerized state is the unwinding of the first part of helix 5 of Tah1, and on comparing a variety of monomeric Tah1 structures this region shows significant structural variability and appears to be rather metastable. The metastable nature at the beginning of the fifth α-helix of Tah1 appears to be an inherent property of this domain and is probably essential for initiating the helix swap. It also appears from our analysis that it is very unlikely that the helix-swapped conformation was induced by a crystallo­graphic contact. The loop connecting helices 4 and 5 of Tah1 makes a very poor crystallization interface and the stability of this interface appears to be very much weaker than the apparent biologically relevant interactions formed by helix 5 in the swapped position. Taken together, these observations provide compelling evidence that dimerization of Tah1 forms the biologically active state that is able to prevent mixed TPR complexes from forming.

Other TPR domain-binding proteins have also been seen to form dimers, but the precise mechanism varies. For example, the C-terminal TPR domains of Sti1 have long been known to be responsible, at least in part, for the dimerization of this protein (Prodromou *et al.*, 1999 ▶). However, molecular details of this dimerization have not been forthcoming. Furthermore, the TPR domain of Sgt1 (suppressor of G2 allele SKP1, not to be confused with Sgt2 and SgtA) has also been shown to homodimerize, although structural detail of this is also lacking (Nyarko *et al.*, 2007 ▶). In contrast, Sgt2 and SgtA (small glutamine-rich TPR-containing protein) have an N-terminal dimerization domain (Tung *et al.*, 2013 ▶; Liou & Wang, 2005 ▶; Chartron *et al.*, 2012 ▶; Tobaben *et al.*, 2003 ▶), as does the orthologue from *Caenorhabditis elegans* (Worrall *et al.*, 2008 ▶), for which structural details of the dimerization have recently been reported (Chartron *et al.*, 2012 ▶). These differ from those of Tah1 and suggest that many different mechanisms of dimerization may be employed by TPR domain-containing proteins.

We also considered testing the dimerization interface by site-directed mutagenesis as a means to test the observed dimeric state of Tah1 when bound to Hsp90. However, because the swapped helices are placed in exactly the same positions as if they had not been swapped, theoretically dimerization of Tah1 can still occur without a helix swap occurring. Essentially the same dimeric state is formed except for some additional interactions with the Hsp90 peptide that result from the uncoiling of the fifth helix of Tah1 that allows helix swapping. However, the additional interactions seen in the Tah1 swapped dimer might lead to increased stability and thus a more stable dimer than one that had not undergone helix swapping. The fact that both dimeric states are essentially the same prevents the helix-swap model from being directly tested by most simple biochemical techniques, including mutagenesis. However, the arrangement of the helix-swapped conformation is fundamentally more stable than one in which the helices have not undergone exchange. Swapping of the helices requires that two interfaces, one between the fifth helices and the other between helices 4 and 5 of the neighbouring molecule, are disrupted in order for the dimer to separate into monomers. However, in a straightforward dimer situation in which the helices have not swapped, only the interface between the fifth helices needs to be disrupted. The extra stability offered in a helix-swapped dimer argues, from a thermodynamic point of view, in favour of the helix-swapped structure being the relevant biological dimer and possibly explains why this conformation was observed in the crystals.

Ultimately, the dimerization of Tah1, whether by helix swapping or not, does explain how a predominantly monomeric TPR protein (in solution) prevents mixed TPR–Hsp90–Tah1 complexes, such as Tah1–(Hsp90)_2_–(Cpr6 or AIP) from forming (Pal *et al.*, 2014 ▶). Furthermore, it explains how the normal hydrophobic pocket that accepts Met3 of the Hsp90 peptide is reformed in line with the human homologue Spag/RPAP3. Whether homodimerization of TPR domain-containing proteins is a universal mechanism that controls and prevents the formation of mixed TPR domain-containing protein complexes with Hsp90 remains to be seen.

## Supplementary Material

PDB reference: Tah1, complex with Hsp90 peptide, 4cgq

Supplementary Figure S1.. DOI: 10.1107/S1399004715004551/dw5132sup1.pdf

## Figures and Tables

**Table 1:** Crystallographic statistics for Tah1SRMEEVD Values in parentheses are for the highest resolution shell.

Data-collection statistics	
Unit-cell parameters	
*a* ()	62.79
*b* ()	62.79
*c* ()	57.90
, , ()	90, 90, 90
Space group	*P*4_3_2_1_2
Wavelength ()	1.5419
Resolution limits ()	13.82.00 (2.052.00)
No. of observations	8230 (590)
Completeness (%)	99.7 (100.0)
Multiplicity	10.3 (10.0)
*R*_merge_	0.098 (0.481)
*R*_p.i.m._(*I*)	0.060 (0.223)
Mean *I*/(*I*)	7.9 (3.0)
Refinement	
Resolution range ()	13.82.00
*R*_cryst_	0.1628
*R*_free_	0.2102
No. of protein atoms	806
No. of ligand atoms	1
No. of solvent atoms	98
Mean *B* factor (^2^)	
Protein	20.8
Ligands	15.3
Solvent	34.0
R.m.s.d., bond lengths ()	0.019
R.m.s.d., bond angles ()	1.791

## References

[bb1] Ali, M. M., Roe, S. M., Vaughan, C. K., Meyer, P., Panaretou, B., Piper, P. W., Prodromou, C. & Pearl, L. H. (2006). *Nature (London)*, **440**, 1013–1017. 10.1038/nature04716 PMC5703407 16625188

[bb2] Back, R., Dominguez, C., Rothé, B., Bobo, C., Beaufils, C., Moréra, S., Meyer, P., Charpentier, B., Branlant, C., Allain, F. H. & Manival, X. (2013). *Structure*, **21**, 1834–1847. 10.1016/j.str.2013.07.024 24012479

[bb3] Battye, T. G. G., Kontogiannis, L., Johnson, O., Powell, H. R. & Leslie, A. G. W. (2011). *Acta Cryst.* D**67**, 271–281. 10.1107/S0907444910048675 PMC3069742 21460445

[bb4] Boulon, S., Pradet-Balade, B., Verheggen, C., Molle, D., Boireau, S., Georgieva, M., Azzag, K., Robert, M. C., Ahmad, Y., Neel, H., Lamond, A. I. & Bertrand, E. (2010). *Mol. Cell*, **39**, 912–924. 10.1016/j.molcel.2010.08.023 PMC4333224 20864038

[bb5] Chartron, J. W., VanderVelde, D. G. & Clemons, W. M. Jr (2012). *Cell. Rep.* **2**, 1620–1632. 10.1016/j.celrep.2012.10.010 PMC3654831 23142665

[bb6] Eckert, K., Saliou, J. M., Monlezun, L., Vigouroux, A., Atmane, N., Caillat, C., Quevillon-Chéruel, S., Madiona, K., Nicaise, M., Lazereg, S., Van Dorsselaer, A., Sanglier-Cianférani, S., Meyer, P. & Moréra, S. (2010). *J. Biol. Chem.* **285**, 31304–31312. 10.1074/jbc.M110.138263 PMC2951205 20663878

[bb7] Emsley, P. & Cowtan, K. (2004). *Acta Cryst.* D**60**, 2126–2132. 10.1107/S0907444904019158 15572765

[bb8] Forget, D., Lacombe, A. A., Cloutier, P., Al-Khoury, R., Bouchard, A., Lavallee-Adam, M., Faubert, D., Jeronimo, C., Blanchette, M. & Coulombe, B. (2010). *Mol. Cell. Proteomics*, **9**, 2827–2839. 10.1074/mcp.M110.003616 PMC3002788 20855544

[bb9] Gonzales, F. A., Zanchin, N. I., Luz, J. S. & Oliveira, C. C. (2005). *J. Mol. Biol.* **346**, 437–455. 10.1016/j.jmb.2004.11.071 15670595

[bb10] Hořejší, Z., Takai, H., Adelman, C. A., Collis, S. J., Flynn, H., Maslen, S., Skehel, J. M., de Lange, T. & Boulton, S. J. (2010). *Mol. Cell*, **39**, 839–850. 10.1016/j.molcel.2010.08.037 20864032

[bb11] Itsuki, Y., Saeki, M., Nakahara, H., Egusa, H., Irie, Y., Terao, Y., Kawabata, S., Yatani, H. & Kamisaki, Y. (2008). *FEBS Lett.* **582**, 2365–2370. 10.1016/j.febslet.2008.05.041 18538670

[bb12] Izumi, N., Yamashita, A., Hirano, H. & Ohno, S. (2012). *Cancer Sci.* **103**, 50–57. 10.1111/j.1349-7006.2011.02112.x PMC11164146 21951644

[bb13] Jiménez, B., Ugwu, F., Zhao, R., Ortí, L., Makhnevych, T., Pineda-Lucena, A. & Houry, W. A. (2012). *J. Biol. Chem.* **287**, 5698–5709. 10.1074/jbc.M111.287458 PMC3285342 22179618

[bb14] Kakihara, Y. & Houry, W. A. (2012). *Biochim. Biophys. Acta*, **1823**, 101–107. 10.1016/j.bbamcr.2011.08.016 21925213

[bb15] Krissinel, E. B., Winn, M. D., Ballard, C. C., Ashton, A. W., Patel, P., Potterton, E. A., McNicholas, S. J., Cowtan, K. D. & Emsley, P. (2004). *Acta Cryst.* D**60**, 2250–2255. 10.1107/S0907444904027167 15572778

[bb16] Kurokawa, M., Zhao, C., Reya, T. & Kornbluth, S. (2008). *Mol. Cell. Biol.* **28**, 5494–5506. 10.1128/MCB.00265-08 PMC2519729 18591256

[bb17] Liou, S.-T. & Wang, C. (2005). *Arch. Biochem. Biophys.* **435**, 253–263. 10.1016/j.abb.2004.12.020 15708368

[bb18] Liu, Y. & Eisenberg, D. (2002). *Protein Sci.* **11**, 1285–1299. 10.1110/ps.0201402 PMC2373619 12021428

[bb19] Morgan, R. M., Hernández-Ramírez, L. C., Trivellin, G., Zhou, L., Roe, S. M., Korbonits, M. & Prodromou, C. (2012). *PLoS One*, **7**, e53339. 10.1371/journal.pone.0053339 PMC3534021 23300914

[bb20] Murshudov, G. N., Skubák, P., Lebedev, A. A., Pannu, N. S., Steiner, R. A., Nicholls, R. A., Winn, M. D., Long, F. & Vagin, A. A. (2011). *Acta Cryst.* D**67**, 355–367. 10.1107/S0907444911001314 PMC3069751 21460454

[bb21] Nagradova, N. K. (2002). *Biochemistry*, **67**, 839–849. 10.1023/a:1019958402194 12223084

[bb22] Nyarko, A., Mosbahi, K., Rowe, A. J., Leech, A., Boter, M., Shirasu, K. & Kleanthous, C. (2007). *Biochemistry*, **46**, 11331–11341. 10.1021/bi700735t 17877371

[bb23] Pal, M., Morgan, M., Phelps, S. E., Roe, S. M., Parry-Morris, S., Downs, J. A., Polier, S., Pearl, L. H. & Prodromou, C. (2014). *Structure*, **22**, 805–818. 10.1016/j.str.2014.04.001 PMC4058522 24794838

[bb24] Pearl, L. H., Prodromou, C. & Workman, P. (2008). *Biochem. J.* **410**, 439–453. 10.1042/BJ20071640 18290764

[bb25] Polier, S., Samant, R. S., Clarke, P. A., Workman, P., Prodromou, C. & Pearl, L. H. (2013). *Nature Chem. Biol.* **9**, 307–312. 10.1038/nchembio.1212 PMC5695660 23502424

[bb26] Prodromou, C. (2012). *Biochim. Biophys. Acta*, **1823**, 614–623. 10.1016/j.bbamcr.2011.07.020 PMC3793855 21840346

[bb27] Prodromou, C., Siligardi, G., O’Brien, R., Woolfson, D. N., Regan, L., Panaretou, B., Ladbury, J. E., Piper, P. W. & Pearl, L. H. (1999). *EMBO J.* **18**, 754–762. 10.1093/emboj/18.3.754 PMC1171168 9927435

[bb28] Roe, S. M., Ali, M. M., Meyer, P., Vaughan, C. K., Panaretou, B., Piper, P. W., Prodromou, C. & Pearl, L. H. (2004). *Cell*, **116**, 87–98. 10.1016/s0092-8674(03)01027-4 14718169

[bb29] Samarsky, D. A., Fournier, M. J., Singer, R. H. & Bertrand, E. (1998). *EMBO J.* **17**, 3747–3757. 10.1093/emboj/17.13.3747 PMC1170710 9649444

[bb30] Siligardi, G., Panaretou, B., Meyer, P., Singh, S., Woolfson, D. N., Piper, P. W., Pearl, L. H. & Prodromou, C. (2002). *J. Biol. Chem.* **277**, 20151–20159. 10.1074/jbc.M201287200 11916974

[bb31] Takai, H., Xie, Y., de Lange, T. & Pavletich, N. P. (2010). *Genes Dev.* **24**, 2019–2030. 10.1101/gad.1956410 PMC2939364 20801936

[bb32] Te, J., Jia, L., Rogers, J., Miller, A. & Hartson, S. D. (2007). *J. Proteome Res.* **6**, 1963–1973. 10.1021/pr060595i 17348703

[bb33] Tobaben, S., Varoqueaux, F., Brose, N., Stahl, B. & Meyer, G. (2003). *J. Biol. Chem.* **278**, 38376–38383. 10.1074/jbc.M301558200 12878599

[bb34] Tung, J.-Y., Li, Y.-C., Lin, T.-W. & Hsiao, C.-D. (2013). *Acta Cryst.* D**69**, 2081–2090. 10.1107/S0907444913019379 24100326

[bb35] Vaughan, C. K., Gohlke, U., Sobott, F., Good, V. M., Ali, M. M., Prodromou, C., Robinson, C. V., Saibil, H. R. & Pearl, L. H. (2006). *Mol. Cell*, **23**, 697–707. 10.1016/j.molcel.2006.07.016 PMC5704897 16949366

[bb36] Worrall, L. J., Wear, M. A., Page, A. P. & Walkinshaw, M. D. (2008). *Biochim. Biophys. Acta*, **1784**, 496–503. 10.1016/j.bbapap.2007.12.003 18187053

[bb37] Zhang, M., Windheim, M., Roe, S. M., Peggie, M., Cohen, P., Prodromou, C. & Pearl, L. H. (2005). *Mol. Cell*, **20**, 525–538. 10.1016/j.molcel.2005.09.023 16307917

[bb38] Zhao, R., Davey, M., Hsu, Y.-C., Kaplanek, P., Tong, A., Parsons, A. B., Krogan, N., Cagney, G., Mai, D., Greenblatt, J., Boone, C., Emili, A. & Houry, W. A. (2005). *Cell*, **120**, 715–727. 10.1016/j.cell.2004.12.024 15766533

