# Supplementary material for: Tah1 helix-swap dimerization prevents mixed Hsp90 co-chaperone complexes
Source: Acta Crystallogr D Biol Crystallogr. 2015 Apr 25;71(Pt 5):1197–206. doi: 10.1107/S1399004715004551 (PMC4427203; doi:10.1107/S1399004715004551)
Supplement: Supplementary file 1 [file d-71-01197-sup1.pdf]

BIOLOGICAL
CRYSTALLOGRAPHY

Volume 71 (2015)

Supporting information for article:

Tah1 helix-swap dimerization prevents mixed Hsp90 co-chaperone complexes

Rhodri M. L. Morgan, Mohinder Pal, S. Mark Roe, Laurence H. Pearl and Chrisostomos Prodromou

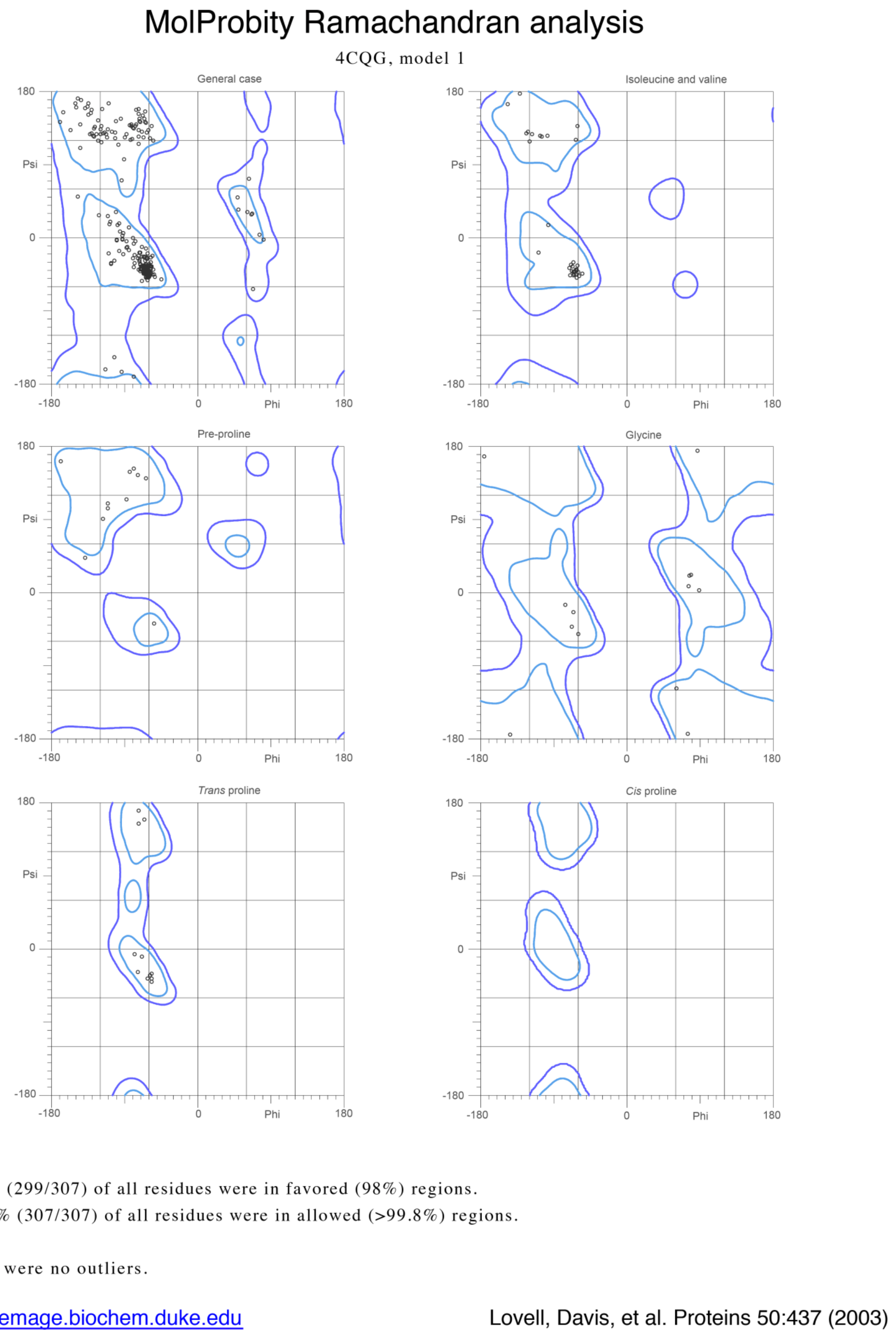

Figure S1 Ramachandran plot.
